# CO-CREATING WITH OLDER PEOPLE CULTURALLY SENSITIVE INCLUSION/EXCLUSION MEASURES

**DOI:** 10.1093/geroni/igac059.469

**Published:** 2022-12-20

**Authors:** Charles Waldegrave, Chris Cunningham, Catherine Love, Monica Mercury

**Affiliations:** Family Centre Social Policy Research Unit, Lower Hutt, Wellington, New Zealand; Research Centre for Maori Health and Development, Wellington, Wellington, New Zealand; Family Centre Social Policy Research Unit, Wellington, Wellington, New Zealand; Family Centre Social Policy Research Unit, Lower Hutt, Wellington, New Zealand

## Abstract

This paper demonstrates a research approach to co-creating social and wellbeing indicator scales through a qualitative methodology, and then applying them in survey format to develop quantitative statistical correlation and regression analysis. We have been co-creating with older Māori (indigenous New Zealanders) a range of scales that more adequately address their notions of wellbeing, social connection, loneliness, discrimination, abuse, and neighbourhood wellbeing than the standard universal scales commonly used in most research. They are designed to be much more inclusive of participants’ worldview and provide more authentic and accurate evidence for policy making and service provision. They draw on the knowledge and experience of the participant communities and reflect their understanding of the indicators. We compare the results with the more common universal indicators and highlight both the universal aspects of each indicator and the culturally specific features to provide more fine-tuned results for policy making and service provision.

